# 

**Published:** 1858-02

**Authors:** 


					﻿No. IX.]
FEBRUARY.
[Vol. xiit.
BUFFALO
MEDICAL JOURNAL
AND

or
MEDICAL AND SURGICAL SCIENCE.
SANFORD I!. HUNT, AL D.,
EDITOR.
AUSTIN FLINT, Jr., M. D.,
ASSOCIATE EDITOR.
BUFFALO, X. Y.:
E. R. J E W ETT <fc CO.’S SI E A M P R ESS.
Commercial Vlv»rtl«er BuiUlitv/H.
185s.
Price 82.00 in advance, or $2.50 at the end of three months
				

